# Smartwatch-Derived Data and Machine Learning Algorithms Estimate Classes of Ratings of Perceived Exertion in Runners: A Pilot Study

**DOI:** 10.3390/s20092637

**Published:** 2020-05-05

**Authors:** Padraig Davidson, Peter Düking, Christoph Zinner, Billy Sperlich, Andreas Hotho

**Affiliations:** 1 Data Science, Institute for Computer Sciences, University of Würzburg, 97074 Würzburg, Germany; hotho@informatik.uni-wuerzburg.de; 2 Integrative and Experimental Exercise Science & Training, Institute for Sport Sciences, University of Würzburg, 97082 Würzburg, Germany; billy.sperlich@uni-wuerzburg.de; 3 Department of Sport, University of Applied Sciences for Police and Administration of Hesse, 65199 Wiesbaden, Germany; Christoph.Zinner@hfpv-hessen.de

**Keywords:** artificial intelligence, endurance, exercise intensity, precision training, prediction, wearable

## Abstract

The rating of perceived exertion (RPE) is a subjective load marker and may assist in individualizing training prescription, particularly by adjusting running intensity. Unfortunately, RPE has shortcomings (e.g., underreporting) and cannot be monitored continuously and automatically throughout a training sessions. In this pilot study, we aimed to predict two classes of RPE (≤15 “Somewhat hard to hard” on Borg’s 6–20 scale vs. RPE >15 in runners by analyzing data recorded by a commercially-available smartwatch with machine learning algorithms. Twelve trained and untrained runners performed long-continuous runs at a constant self-selected pace to volitional exhaustion. Untrained runners reported their RPE each kilometer, whereas trained runners reported every five kilometers. The kinetics of heart rate, step cadence, and running velocity were recorded continuously ( 1Hz) with a commercially-available smartwatch (Polar V800). We trained different machine learning algorithms to estimate the two classes of RPE based on the time series sensor data derived from the smartwatch. Predictions were analyzed in different settings: accuracy overall and per runner type; i.e., accuracy for trained and untrained runners independently. We achieved top accuracies of 84.8% for the whole dataset, 81.8% for the trained runners, and 86.1% for the untrained runners. We predict two classes of RPE with high accuracy using machine learning and smartwatch data. This approach might aid in individualizing training prescriptions.

## 1. Introduction

Several objective (e.g., heart rate, distance covered, velocity, accelerations) and subjective load parameters (e.g., ratings of perceived exertion) exist to estimate a runner’s physiological response to an exercise stimulus. Recent articles provide an overview of objective and subjective load markers [1,2]. Subjective load markers may trump objective markers while being simple and inexpensive to assess for estimating a runner’s load [3,4].

The rating of perceived exertion (RPE) [5] is a widely explored and frequently applied subjective parameter. It indicates how hard or easy a subject perceives an exercise task based on feed-forward mechanisms stemming from, e.g., thermal, cardiorespiratory, or metabolic stimuli and is modulated by psychological factors (e.g., previous experience, understanding of the task etc.) [6]. While different RPE scales exist, the 6–20 Borg Scale is the most frequently employed scale in exercise science [5,6].

Independently of gender, age, and fitness status, RPE is closely related to metabolic (blood lactate concentration, r=0.83) and cardiac (heart rate, r=0.74) stress parameters [7]. An RPE value of 14 corresponds to the second lactate threshold (maximal lactate steady state) [7], a benchmark which distinguishes between so called “threshold training” (RPE <14) and high-intensity exercise (RPE >15) [8].

It is well known that exercise, depending on the intensity domain, stimulates physiological adaptations [9]. By predetermining RPE ranges for these domains and exercising within these ranges, a runner can easily target and avoid certain intensities, depending on the specific training goal. However, assessing RPE in daily training routines is difficult due to lack of compliance, burdens of individual recall bias [10], poor knowledge on how to report RPE, peer pressure, and dishonest reporting to influence and manipulate future training prescriptions [11].

In the case of RPE-reporting, coaches would benefit from wearable-derived and machine-learning predicted classes of RPE to overcome the aforementioned limitations in order to (i) verify a runner’s rating during exercise and (ii) adjust exercise intensity. In other contexts, machine learning algorithms based on wearable-derived time series data already allow one to predict other load markers (e.g., heart rate response [12]). So far, there is no study which has applied data collected with low cost, simple, and commercially-available sensors to predict a runner’s RPE during long and exhaustive runs.

Here, we aimed to (i) estimate classes of RPE (i.e., ≤15 “Somewhat hard to hard” on 6–20 Borg scale vs. RPE >15 via machine learning models which were based on the data obtained by easy-to-use and commercially-available smartwatches in untrained and trained runners; and (ii) evaluate which machine learning approach reveals the highest accuracy for predicting the two classes.

## 2. Methods

### 2.1. Participants

Twelve male runners participated in our study. These runners were separated into two groups based on their peak oxygen consumption (VO_2 peak_): six runners (height: 181.6 ± 8.3 cm, body mass: 74.9 ± 8.9 kg, age: 32.4 ± 9.1 years, VO_2 peak_: 60.9 ± 6.6 mL min^−1^ kg^−1^) were classified as trained runners, and the remaining six as recreational runners (height: 178.0 ± 6.0 cm, body mass: 77.0 ± 4.2 kg, age: 26.3 ± 1.5 years, VO_2 peak_: 49.0 ± 3.5 mL min^−1^ kg^−1^). All participants gave their consent to participate in this study after being informed about all testing procedures as well as the potential risks and benefits involved. The local university’s ethical committee pre-approved the study design, which was in accordance with the Declaration of Helsinki.

### 2.2. Experimental Design

Each participant reported to the testing facilities twice. On the first visit, all participants performed a ramp like protocol to determine VO_2 peak_ and maximal heart rate. The protocol was enacted on an outdoor 400 m track starting with 5 min at 7 km h^−1^, followed by increases of 1 km h^−1^ min^−1^. Gases were measured by a portable breath-by-breath gas analyzer (MetaMax 3b, Cortex, Leipzig, Germany), whereas heart rate was measured by a GPS-enabled heart rate watch (V800, Polar Electro Oy, Kempele, Finland).

On the second visit (at least 72 h after the first visit) all participants performed a long continuous submaximal run wearing the GPS-enabled heart rate watch (sampling of all data at 1 Hz) after a standardized 10 min warm-up. All participants ran at a self-selected but constant velocity without applying a pacing strategy until full exhaustion on a predefined, flat, outdoor course. The running velocity was monitored and controlled with GPS.

All runners reported their RPE, ranging from 6–20, to the an investigator, who was following the runner on a bicycle. The trained runners ran a flat 5 km-course and reported their RPE scores after each full round. The untrained runners ran a flat 2 km-course and reported their RPE scores each kilometer. Table 1 shows additional statistics about the collected scores.

### 2.3. Data Analysis

This pilot study incorporated several models for predicting two classes of RPE from multivariate time series during moderate-intensity continuous running. Models included are two baselines (majority vote and Borg’s classification), three classifiers using distance metrics to calculate similarities (symbolic Fourier approximation [13,14], k-nearest neighbors [15], and SVM [16] with specialized kernels), and two types of neural networks (CNN [17] and GRU [18]). The RPE labels were grouped into two classes of subjective ratings of running intensity:

- “Somewhat hard to hard” (non-exerted): RPE ≤15.
- (exerted): RPE >15.

**Figure 1:**
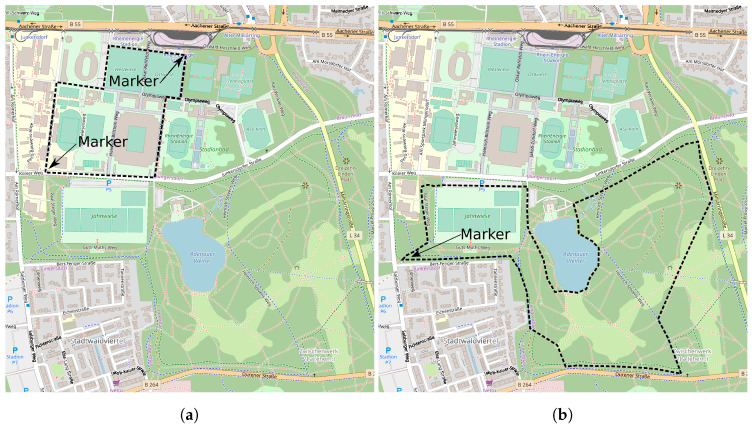
Map view of the tracks for both study groups. (**a**) Map view of the 2 km track for the untrained runners., (**b**) Map view of the 5 km track for the trained runners. Map views were created using ©OpenStreetMap contributors.

Since the runners performed the submaximal runs as an open-end task, we labeled all RPE ≤15 as “Somewhat hard to hard” and >15 as at specific time points (“markers” in Figure 1); e.g., $l_{t,r}$ = runner *r* at his respective round and time *t*.

Therefore, each machine learning algorithm was targeted to predict a label ${\hat{l}}_{t,r}$ for a certain part of the run as close as possible to the originally given label. We measured the discrepancy between each prediction and original label using a loss function (e.g., cross-entropy). The task was defined as follows:

$$\min\sum_{r\in \mathrm{Runners}}\sum_{t\in \mathrm{Markers}(r)}\mathrm{loss}\left(l_{t,r},{\hat{l}}_{t,r}\right).$$

Each algorithm was given a multivariate time series and the ground truth $l_{t,r}$ for the corresponding round. Within the multivariate time series classification, we aimed to include the raw values of each sensor (i.e., heart rate, cadence, and velocity), and their interactions. In a preliminary analysis we found feature extraction not to yield better results than using the raw sensor values.

### 2.4. Machine Learning Classifiers

The parameter settings for all following classifiers are summarized in Table A1 in the Appendix A. Hyper-parameters were optimized using random search [19].

#### 2.4.1. Baselines

We used two baselines: a majority vote and a baseline derived from Borg. The majority vote always predicts the most common class for a segment; i.e., “Somewhat hard to hard”.

Our second baseline follows Borg’s classification of the perceived exertion and the heart rate [5]: RPE≃HR/10. We used the average heart rate of the given time series segment and divided it by ten, resulting in a RPE score, which was used to assign a suitable label.

#### 2.4.2. Distance Based Classifiers

Many classic machine learning classifiers need to measure the distance between two given samples, in order to estimate their relative closeness. To this end, one can use a metric. With this similarity measure it is possible to classify input data to its closest known training sample(s). We used symbolic Fourier approximation, *k*-nearest neighbor classifiers, and support vector machines to classify levels of exertion by distance measuring.

**Symbolic Fourier approximation:** Symbolic Fourier approximation (SFA) [13] transforms the given time series into the frequency domain. After that, a low-pass filter (smoothing) is applied and the series is quantized into words via multiple coefficient binning ([13], Figure 2). Those words are used to measure distances between input data using common string algorithms. The quality of approximation is regulated by the number of applied Fourier coefficients. SFA allows one to adjust this by three tunable parameters: the minimum number of symbols, representing the time series (MinF); the maximum number of symbols (MaxF); and the maximum number of available symbols (MaxS). We want to classify multivariate time series; therefore, the “WEASEL+MUSE” [14] algorithm was used in our work.

**Nearest neighbors classifier:** Nearest neighbors classifiers are voting-based classifiers. A special case of this supertype is the *k*-nearest neighbors classifier (kNN) [15]. For any given time series, this classifier searches for the *k* nearest time series according to a distance function or metric. Classification labels are assigned via a majority vote within this neighborhood. Prominent metrics for directly measuring distances between multivariate time series include dynamic time warping (DTW) [20], the (squared) euclidean distance, and the cityblock (manhattan) distance. We used DTW as a metric, since it showed the best results. In the simplest case, k=1, the label of the closest reference time series is used. With increasing *k*, the locality aspect of this classifier is superseded and lastly diminished to the majority vote baseline, if k≥|Labels|. Additionally, one can weigh the votes of the neighborhood by their respective weights; i.e., the further a neighbor is, the less it contributes to the majority vote.

**Support vector machine:** Support vector machines (SVM) [16] belong to the class of supervised learning models. To classify input data, the SVM finds a hyperplane that maximizes the distance between instances of different labels, whereas instances of the same class remain on the same side of this plane. This plane is also called the separating hyperplane. Nonlinear classification is achieved using a kernel function, which additionally enables classification of multivariate time series [21]. Since some points may still be non-separable, a penalty parameter (C) is introduced. If the parameter is too small, the support vector machine tends to underfit, whereas too large a value leads to a hard margin between both classes.

#### 2.4.3. Neural Networks

Another class of classifiers employed in the present investigation was the neural network class. Due the multivariate nature of the collected time series, we considered 1D-convolutional neural networks (CNN) and gated recurrent units (GRU) [18], because they are well suited for this kind of data. We chose GRU over long-short term networks (LSTM), as their performances are similar. Additionally, in smaller datasets, GRU showed superior performance [18]. We selected only one recurrent neural network for this pilot study.

**Gated recurrent units:** Recurrent neural networks (RNNs) extend feed forward networks by introducing feedback connections within layers. This makes RNNs especially suited to handle sequential data such as time series, as preceding time stamps are included into the calculations with decaying weight. The most influential parameters of the GRU are the number of units (neurons) in use and the output function of each unit. Furthermore, we can regulate the linear and recurrent dropout [22] within each unit. Dropout was introduced to help reduce the problem of overfitting. When using too few units, the model is prone to underfitting, whereas too many units tend to lead to overfitting. Additionally the number of trainable parameters (weights and biases) increases drastically with more units and deeper networks.

**Convolutional neural networks:** In a convolutional neural network, a filter (matrix) is convolved with the input matrix. Each filter functions as a feature extractor. The higher the number of filters, the more feature maps generated, which regulates the number of output weights. After each convolution, these filters are moved across the input by a fixed number of elements (strides) in order to detect their specific features at different locations. After each layer of convolution, we use an intermediate batch normalization layer [23]. Batch normalization assists convolutional neural networks to converge more quickly and improve generalization. Lastly, we tested the ReLU and the SeLU activation functions. Ramifications regarding the numbre of filters remain the same as in the case of the GRU. Therefore, deeper networks with smaller numbers of filters are favored when using small datasets. In contrast to [17], we used *global maximum pooling instead of global average pooling*.

For all neural networks, we employed the *Adam* optimizer with the default parameters ($lr=10^{-3}$) and categorical cross-entropy as the loss function. Additionally we used early stopping with a patience of 10 while training for 100 epochs.

## 3. Evaluation of Classifiers

We applied standard metrics for classification results in accordance to [24] (Chapter 8.3). Those are the overall accuracy, precision, and recall, for each class individually and globally weighted by support. Additionally, we included the $F_{1}$ score to capture the overall performance in one metric [25].

### 3.1. Model Training Procedure

Since the main objective of the present study was to identify a generalizing model for assigning RPE labels to given multivariate time series, we employed repeated k-fold cross validation [26]. We chose k=6 for our experiments and additionally set the constraint on any fold to contain one runner from each group.

**Figure A1:**
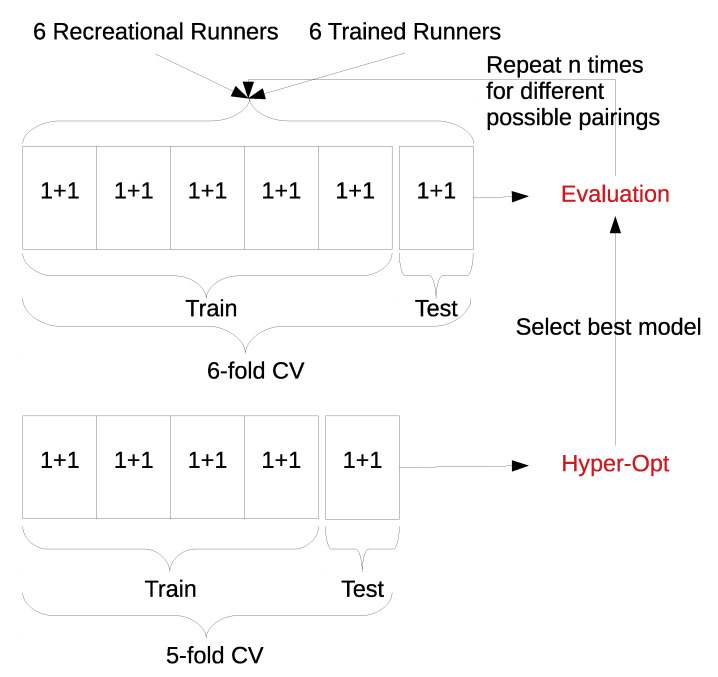
Visual representation of the training and evaluation procedure.

For each test-fold, we used the remaining runners for a hyper-parameter search in a five-fold cross-validation with the same constraints on the validation sets within the search. After that, we employed the best model found by averaging the accuracies of the five folds on the test-fold. This procedure is visualized in Figure A1 in the Appendix B. All time series were scaled per runner via standard scaling (z-normalization).

We developed neural networks using *keras* [27] with the *tensorflow* [28] back-end. To increase the number of training samples, we applied the following augmentation technique: for every exertion label $l_{t,i}$ at time stamp *t* and runner *r*, consider a maximal temporal offset *mo* within which the given score can be considered constant. That is, for all augmented labels $l^{\prime }$, the following equivalence holds true:

$$l_{t^{\prime },r}^{\prime }=l_{t,r}\;|\;\forall t^{\prime }:t-\mathrm{mo}\leq t^{\prime }\leq t+\mathrm{mo}$$

With that, we labeled windows of length L∈ (30, 60, 90 and 120) s, such that each classifier was handed input data from $R^{L\times 3}$, for the three input features, heart rate, velocity, and cadence. Briefly, we extracted every possible subsequent multivariate time series of length *L* within the described window. We experimented with different values of the *mo* parameter, but found *mo* = 180 s to be a fair trade-off. The value was large enough to generate a feasible amount of data available to train a neural network, yet not so large that i) two succinct labels overlapped or ii) the assumption of a constant fatigue score within that interval was void. A window length of *L* = 60 s showed the best results for our setting; therefore, we only report classification accuracies using this observation length. Using this combination (*L* = 60 s, *mo* = 180 s) yields 20,000 training samples in the 5-fold cross-validation and 22,000 within the evaluation setting.

### 3.2. Significance Testing

For significance testing, we used McNemar’s test [29] with a significance level of α=5%, and defined the following hypotheses:

- $H_{0}$: Both classifiers perform equally well.
- $H_{\alpha }$: Classifier 2 performs significantly better than classifier 1 with significance level α.

Repeated (*n* times) k-fold cross validation was used. Overall there were 6! = 720 (recall the definition of a fold given in Section 3.1) possible repetitions of which we used n=15 for our experiments. For every label from each runner, we employed the most common as the "real" prediction for significance testing. In order to avoid draws in votes, we chose an odd number of runs.

With these predictions, we built the required contingency table $C\in N^{2\times 2}$ with the following steps:

- $C_{0,0}$ = Correct predictions made by both classifiers.
- $C_{1,1}$ = Incorrect predictions made by both classifiers.
- $C_{0,1}$ = Correct predictions made by classifier 1, but incorrect ones by classifier 2.
- $C_{1,0}$ = Correct predictions made by classifier 2, but incorrect ones by classifier 1.

To calculate the statistical values necessary for comparison with the $\chi^{2}$ distribution, we applied the corrected version as shown in [30]:

$$Z:=\frac{{\left(|C_{0,1}-C_{1,0}|-1\right)}^{2}}{C_{0,1}+C_{1,0}}$$

We rejected the null-hypothesis with an error of α if, and only if, the statistical value was greater than the reference value of the $\chi^{2}$ distribution with one degree of freedom, $\chi_{1,1-\alpha }^{2}$. With α=5%, this reference value is $\chi_{1,0.95}^{2}=3.841459$.

## 4. Results

### 4.1. All Runners

The trained runners ran 27.5 ± 3.3 km at an average pace of 78% HF_max_. The untrained runners ran 13.1 ± 2.4 km at an average pace of 82 HF_max_. Overall, *n* = 79 RPE values were collected from the untrained runners and *n* = 33 from trained runners.

Table 2 displays all results achieved by the different machine learning classifiers. The $F_{1}$ metrics in Table 2 suggest that only the baselines (i.e., majority vote, Borg’s classification) show a bias towards a certain class.

The accuracies when predicting the classes of RPE were 58.9%, 50.9%, 73.2%, 79.5%, 83.0%, 82.1%, and 84.8% achieved by the majority vote, Borg’s classification, SFA, SVM, DTW+KNN, GRU, and CNN, respectively. The neural networks and the distance based classifiers significantly differ from the majority vote (marked by ♣, P<0.001). CNN and k-NN classifiers are significantly different to the SFA classifier (marked by ♠, P<0.05). All other differences between classifiers are non-significant.

Table 3 displays a detailed confusion matrix for the CNN classifier. Classes of RPE were estimated with accuracies of up to 88.9% and 79.6%, respectively.

### 4.2. Untrained Runners

Table 4 displays selected classification metrics for recreational and trained runners separately.

For recreational runners, RPE classes “Somewhat hard to hard” vs. achieved 60.8%, 41.8%, 72.2%, 82.3%, 86.1%, 83.5%, and 86.1% prediction accuracy by the majority vote, Borg’s classification, SFA, SVM, DTW+KNN, GRU, and CNN, respectively.

SVM, DTW+KNN, GRU, and CNN are significantly different to the majority vote (P<0.01). DTW+KNN and CNN are significantly different from the SFA (P<0.05). All other comparisons between classifiers were non-significant. Table 5 shows the confusion matrix for the CNN for recreational runners.

Both RPE classes were estimated with a precision up to 86.3% and 85.7%, respectively.

### 4.3. Trained Runners

For trained runners, accuracies when predicting the RPE classes “Somewhat hard to hard” vs. were 54.6%, 72.7%, 75.8%, 72.7%, 75.8%, 78.8%, and 81.8% achieved by the majority vote, Borg’s classification, SFA, SVM, DTW+KNN, GRU, and CNN, respectively.

For that group of runners, no classifier is significantly better than the majority vote. The CNN shows the highest accuracy for this runner type.

As revealed by the $F_{1}$ score in Table 4 for trained runners only the majority vote yields unbalanced predictions and is biased towards the class.

Table 6 shows the confusion matrix for the CNN in the trained runner setting.

RPE classes “Somewhat hard to hard” and can be estimated with precision of up to 100.0% and 71.4%, respectively.

## 5. Discussion

In this study, we aimed to estimate two classes of RPE (“Somewhat hard to hard” (RPE ≤15) vs. (RPE >15)) in untrained and trained runners. We used this threshold to formulate a classification task for our machine learning algorithms. Due to the sparsity of different RPE levels, we refrained from considering a regression task for this pilot study. Furthermore, we evaluated whether both RPE domains can be predicted with data derived by low-cost, commercially-available smartwatches. The present results show that classes of RPE are predictable with an accuracy of 86 by employing convolutional neural networks.

Currently, little research exists on estimating RPE domains in runners using wearable sensors and machine learning algorithms. To the best of our knowledge, this is the first study using data derived by easy-to-use, low-cost, and commercially-available wearable sensors to estimate classes of RPE in runners. In a related setting, the authors [31,32] employed a sensor embedded in a shoe and heart rate and biomechanical data to distinguish between three classes of perceived fatigue and achieved an accuracy up to 91.8% for an individual during an one hour run. However, their work partly depends on customized (non-commercial) sensors, which impairs the application of their findings in the daily routine work for a broad spectrum of coaches.

The machine learning classifiers in the present investigation achieved varying accuracies for untrained and trained runners. The Borg’s classification achieved 41.8% and 72.7% accuracy for the untrained and trained runners, respectively. Within our extensive data analysis, CNN revealed highest accuracy when predicting the RPE domains, however depending on data density other models such as LSTM networks, might be better suited to predict the two classes. Nevertheless, CNNs have proven to achieve state of the art performance in time series classification. Additionally, they require fewer trainable parameters and are therefore more likely to learn useful features from small datasets.

The distance based classifiers (KNN and SVM), achieved accuracies of 79.5% and 83.0% for untrained and trained runners. The lower accuracy indicates that the additional parameters (cadence and velocity) lessen the strong tendency of the heart rate for high levels of perceived exertion in untrained runners; hence they contain additional information to predict the two RPE classes. Within the trained runners, this feature seems most dominant for classification since the "Borg classifier" achieves competitive results compared with the other classifiers. In contrast to KNN and SVM, neural networks (NN) show similar classification quality for both runner types. Their classification power remains the same for both performance levels (trained vs. recreational), though with a negligent advantage towards the recreational runners. Based on our analysis ,the prediction of RPE seems dependent on the runner’s fitness level, the integrated data, and the type of analysis.

One limitation of our study is the small amount of collected RPE values. This likely impairs selecting the best machine learning classifier for the given task, as some of these techniques need more input data. Yet, achieving an accuracy of up to 86% using CNN with small amount of input data shows the feasibility to estimate classes of RPE based on data obtained from a commercially-available smartwatch and machine learning classifiers. To improve the accuracy of machine learning algorithms, future work should evaluate the use of individual HR-related intensity thresholds [33].

Our pilot study proves that the limitations of collecting RPE values in the daily work of coaches with athletes (compliance errors, recall bias, peer pressure, dishonest reporting, etc.) may be overcome by data obtained from a commercially-available smartwatch and machine learning classifiers. Future studies should aim to improve the number of classes of RPE which can be estimated. This may enable coaches to prescribe more personalized training by individually adjusting exercise intensity to ensure optimal performance enhancement and to avoid overuse.

## 6. Related Work

To the best of our knowledge, this is the first study using easy-to-use, low-cost, and commercially-available sensors to estimate classes of RPE in trained and untrained runners.

While some use inertial measurement units (IMUs) comprise an accelerometer and a gyroscope, others use customized shoes to assess walking patterns.

Buckley et al. [34] placed IMUs on the lumbar and both shanks of 21 participants. They used random forests for their binary classification from extracted features of both measurements.

Zhang et al. [35] employed support vector machines (SVM) to distinguish fatigued and non-fatigued gait patterns using IMUs and infra-red markers on 17 test subjects.

Both of the aforementioned induced fatigue using different methods (pacer test [34], squatting exercise [35]) and labeled all pre-exercise sequences as non-fatigued, whereas all post-exercise sequences served as fatigued ones, and therefore only indirectly used RPE values given by the subjects.

Eskofier et al. [31,32], in contrast, employed a comparable exercise protocol to ours, as fatigue was induced by the running exercise itself. They used customized shoes to analyze gait patterns, alongside derived features from the heart rate variability, to distinguish between three (two in [32]) fatigue states on a scale from 0 to 6. Their aim was to create a real-time classification system on an embedded micro controller. For that, they collected data from 431 runners (254 usable for classification) and asked each runner every 5 min about their fatigue level. Classification was done via a linear SVM on each step.

## 7. Conclusions

Based on our extensive data analysis, we showed that smartwatch-derived data and machine learning algorithms proved to estimate two classes (i.e., “Somewhat hard to hard” and of RPE with high accuracy. Of all applied algorithms and within our study design, 1D-CNN revealed the highest accuracy when predicting the two classes of RPE in trained and untrained runners.

## Figures and Tables

**Table 1:** Dataset statistics.

Statistic	Untrained Runners	Trained Runners	Overall
Number of RPE values	79	33	112
Number of “Somewhat hard to hard”	48	18	66
Number of “Hard to very hard”	31	15	46
Average number of rounds	6.6	5.5	6.1
Covered Distance (km)	79	165	244
Total Running Time (min)	75.2	110.4	
Time Between Inquiries (min)	6.1	24.3	

**Table 2:** Summary of the achieved classification results. All values were obtained using the weighted (by support) averaging scheme. ♣ marks significant difference to the majority vote. ♠ marks significant difference to SFA. The best results are printed in bold.

Model	Accuracy (%)	Precision (%)	Recall (%)	F_1_ (%)
Majority Vote	58.9	34.7	58.9	43.7
Borg’s classification	50.9	73.1	50.9	43.4
WEASEL+MUSE (SFA)	73.2 ♣	73.2	73.2	73.2
SVM	79.5 ♣	79.4	79.5	79.4
DTW + KNN	83.0 ♣,♠	83.1	83.0	83.1
GRU	82.1 ♣	82.1	82.1	82.1
CNN	**84.8** ♣,♠	85.1	84.8	**84.9**

**Table 3:** Confusion matrix for the CNN classifiers. Each row represents the correct label (e.g., ground truth), whereas the columns display the predictions made by the network.

Accuracy: 84.8%	“Somewhat hard to hard”	“Hard to very hard”	Recall (%)
“Somewhat hard to hard”	56	10	84.8
“Hard to very hard”	7	39	84.8
Precision (%)	88.9	79.6	

**Table 4:** Summary of the achieved classification results grouped by performance levels of the runners. The nomenclature remains the same as in Table 2. Labels were extracted from the overall evaluation procedure, and not each runner type trained separately. ♣ marks significant difference to the majority vote. ♠ marks significant difference to SFA. The best results are printed in bold.

	Untrained Runners		Trained Runners	
Model	Accuracy (%)	$F_{1}$ (%)	Accuracy (%)	$F_{1}$(%)
Majority Vote	60.8	45.9	54.5	38.5
Borg’s classification	41.8	27.4	72.7	72.0
WEASEL+MUSE (SFA)	72.2	72.0	75.8	75.8
SVM	82.3 ♣	82.2	72.7	72.8
DTW + KNN	**86.1** ♣,♠	**86.0**	75.8	75.8
GRU	83.5 ♣	83.4	78.8	78.8
CNN	**86.1** ♣,♠	85.9	**81.8**	**81.5**

**Table 5:** Confusion matrix for the CNN classifiers in the recreational runner setting. Each row represents the correct label (e.g., ground truth), whereas the columns display the predictions made by the network.

Untrained Runners			
Accuracy: 86.1%	“Somewhat hard to hard”	“Hard to very hard”	Recall (%)
“Somewhat hard to hard”	44	4	91.7
“Hard to very hard”	7	24	77.4
Precision (%)	86.3	85.7	

**Table 6:** Confusion matrix for the CNN classifiers in the trained runner setting. Each row represents the correct label (e.g., ground truth), whereas the columns display the predictions made by the network.

Trained Runners			
Accuracy: 81.8%	“Somewhat hard to hard”	“Hard to very hard”	Recall (%)
“Somewhat hard to hard”	12	6	66.7
“Hard to very hard”	0	15	100.0
Precision (%)	100.00	71.4	

## Appendix A. Model Parameter Ranges

Within the hyper-optimization step for model parameter grid search, we used the ranges displayed in Table A1.

**Table A1:** Models and their parameters alongside their specific ranges used within the hyper-optimizing steps.

Model	Parameter	Range
WEASEL+MUSE	MinF	[1, 15]
	MaxF	[MinF, 20]
	MaxS	[4, 20]
DTW+KNN	k	[1, 20]
	weights	[uniform, distance]
	metric	[dtw, euclidean, sqeuclidean, cityblock]
SVM	C	$\left[10^{-10},10^{-9},\ldots ,10^{0},\ldots ,10^{10}\right]$
	degree	[1, 20]
	decision function	[ovo, ovr]
	kernel	[gak, linear, poly, rbf, sigmoid]
GRU	units	[1, 128]
	recurrent dropout	(0, 1)
	linear dropout	(0, 1)
CNN	filters	[1, 256]
	kernel size	[1, 16]
	strides	[1, kernel size]
	activation	[relu, selu]

## Appendix B. Evaluation and Training Procedure

The evaluation and training procedure mentioned in Section 3.1 is displayed in Figure A1.
